# Microglial activation in Parkinson’s disease using [^18^F]-FEPPA

**DOI:** 10.1186/s12974-016-0778-1

**Published:** 2017-01-11

**Authors:** Christine Ghadery, Yuko Koshimori, Sarah Coakeley, Madeleine Harris, Pablo Rusjan, Jinhee Kim, Sylvain Houle, Antonio P. Strafella

**Affiliations:** 1 Neurology Division, Department of Medicine, Morton and Gloria Shulman Movement Disorder Unit & E.J. Safra Parkinson Disease Program, Toronto Western Hospital, UHN, University of Toronto, Ontario, Canada; 2 Research Imaging Centre, Campbell Family Mental Health Research Institute, Centre for Addiction and Mental Health, University of Toronto, Toronto, Ontario Canada; 3 Division of Brain, Imaging and Behaviour – Systems Neuroscience, Krembil Research Institute, UHN, University of Toronto, Ontario, Canada; 4 Toronto Western Hospital and Institute, CAMH-Research Imaging Centre, University of Toronto, Toronto, Ontario M5T 2S8 Canada

**Keywords:** Neuroinflammation, TSPO imaging, PET, Parkinson’s disease

## Abstract

**Background:**

Neuroinflammatory processes including activated microglia have been reported to play an important role in Parkinson’s disease (PD). Increased expression of translocator protein (TSPO) has been observed after brain injury and inflammation in neurodegenerative diseases. Positron emission tomography (PET) radioligand targeting TSPO allows for the quantification of neuroinflammation in vivo.

**Methods:**

Based on the genotype of the rs6791 polymorphism in the TSPO gene, we included 25 mixed-affinity binders (MABs) (14 PD patients and 11 age-matched healthy controls (HC)) and 27 high-affinity binders (HABs) (16 PD patients and 11 age-matched HC) to assess regional differences in the second-generation radioligand [^18^F]-FEPPA between PD patients and HC. FEPPA total distribution volume (*V*_T_) values in cortical as well as subcortical brain regions were derived from a two-tissue compartment model with arterial plasma as an input function.

**Results:**

Our results revealed a significant main effect of genotype on [^18^F]-FEPPA *V*_T_ in every brain region, but no main effect of disease or disease × genotype interaction in any brain region. The overall percentage difference of the mean FEPPA *V*_T_ between HC-MABs and HC-HABs was 32.6% (SD = 2.09) and for PD-MABs and PD-HABs was 43.1% (SD = 1.21).

**Conclusions:**

Future investigations are needed to determine the significance of [^18^F]-FEPPA as a biomarker of neuroinflammation as well as the importance of the rs6971 polymorphism and its clinical consequence in PD.

## Background

Parkinson’s disease (PD), classically presenting with progressive motor symptoms such as tremor, rigidity, bradykinesia, and postural instability, is the second most common neurodegenerative disorder [1]. Neuroinflammatory processes including activated microglia [2, 3] as well as increased concentration of inflammatory cytokines [4–6] have been identified to play an important role in PD.

Translocator protein (TSPO), which is an 18-kDa protein located on outer mitochondrial membranes in microglia, can be found in healthy brains at very low levels. Its increased expression has been observed in various neuropathological conditions such as amyotrophic lateral sclerosis, Alzheimer disease (AD), frontotemporal dementia, and multiple sclerosis [7].

Therefore, TSPO expression may represent a potential in vivo biomarker of neuroinflammation and reactive gliosis [8]. Positron emission tomography (PET) enables the quantification of neuroinflammation by using radioligands that target TSPO [9].

Thus far, several studies have explored the relationship between neuroinflammation and PD by using the first and most widely used radioligand [^11^C]-PK11195 [10, 11]. However, findings are inconclusive, and elevated TSPO binding in nigro-striatal regions have not been consistently observed [11]. This discrepancy might derive from well-known technical limitations of [^11^C]-PK11195 such as low signal-to-noise ratio, high nonspecific binding, low brain penetration, and high plasma protein binding [12]. As a result, second-generation TSPO radioligands such as [^18^F]-FEPPA have been developed to offer a superior quality to quantify TSPO expression in vivo [13]. [^18^F]-FEPPA has shown a high affinity for TSPO, a suitable metabolic profile, high brain penetration, and good pharmacokinetics [14, 15].

Currently, most of the second-generation TSPO radioligands show three patterns of binding affinity based on genetic polymorphism: high-affinity binders (HABs), mixed-affinity binders (MABs), and low-affinity binders (LABs) [16]. These variations in TSPO binding affinity are derived from a single polymorphism located on exon 4 of the TSPO gene (rs6971) [17], which accounts for some of the large inter-individual variability in the outcome measures [16].

The current study represents an extension of our previous work [18] aiming to investigate whether TSPO imaging with [^18^F]-FEPPA using total distribution volume (*V*_T_) as an index of TSPO density, could be used as a potential biomarker for neuroinflammation in PD. In this study, we included a larger sample size to assess if there are regional differences in FEPPA *V*_T_ for cortical as well as subcortical brain regions between PD patients and healthy controls (HC). In addition, we are aiming to confirm our previous results by presenting potential interactions between the rs6791 polymorphism and neuroinflammation in PD.

## Methods

### Participants

Thirty patients meeting UK Brain Bank criteria for the diagnosis of idiopathic PD and 22 healthy controls participated in our study. Exclusion criteria for all participants included (1) history of a head injury, psychiatric or neurological (except PD for the patients) diseases, (2) alcohol or drug dependency or abuse, (3) contraindications for MRI scanning, and (4) use of nonsteroidal anti-inflammatory drugs. All participants were assessed for their cognitive performance using the Montreal Cognitive Assessment (MoCA) as well as their level of depression using the Beck Depression Inventory II (BDI II). For nine HC, cognitive assessment scores were only available from the Mini-Mental State Examination (MMSE). PD patients were also assessed for motor severity of the disease using the motor subset of the Unified Parkinson Disease Rating Scale (UPDRS-III). All participants underwent PET and structural MRI scans. All participants provided written informed consent following full explanation of the study procedures. The study was approved by the Centre for Addiction and Mental Health Research Ethics Board.

### PET data acquisition

A detailed description of [^18^F]-FEPPA synthesis was described in a previous publication [14]. It can be reliably and quickly labeled with [^18^F] by nucleophilic displacement of a tosylate, leaving group in a fast one-step reaction yielding a sterile and pyrogen-free product after purification and formulation.

The PET images were obtained using a 3D High Resolution Research Tomography (HRRT) (CPS/Siemens, Knoxville, TN, USA), which measures radioactivity in 207 slices with an interslice distance of 1.22 mm. A custom-fitted thermoplastic mask was made for each participant and used with a head fixation system during the PET scans to minimize head movement (Tru-Scan Imaging, Annapolis). Following a transmission scan, intravenous [^18^F]-FEPPA was administered as a bolus. The scan duration was 125 min. The images were reconstructed into 34 time frames: 1 frame of variable length until the radioactivity appears in the field of view (FOV), 5 frames of 30 s, 1 frame of 45 s, 2 frames of 60 s, 1 frame of 90 s, 1 frame of 120 s, 1 frame of 210 s, and 22 frames of 300 s.

All PET images were corrected for attenuation using a single photon point source, 137Cs (T50 = 30.2 years, Eγ = 662 keV) and were reconstructed by filtered back projection algorithm using a HANN filter at Nyquist cutoff frequency. The reconstructed image has 256 × 256 × 207 cubic voxels measuring 1.22 × 1.22 × 1.22 mm^3^, and the resulting reconstructed resolution is close to isotropic 4.4 mm, full width at half maximum in plane and 4.5 mm full width at half maximum axially, averaged over measurements from the center of the transaxial FOV to 10 cm off-center in 1.0-cm increments. In addition, for frame realignment for head motion correction, each image was reconstructed without attenuation correction using three iterations of iterative reconstruction [15].

### MRI acquisition

MR images for all the participants were acquired for co-registration with the corresponding PET image and the anatomical delineation of the regions of interest (ROIs). Proton density (PD)-weighted MR images were chosen for better identification of ROIs [19]. 2D oblique PD-weighted MR images were acquired with a General Electric Discovery 3.0 T MRI scanner (slice thickness = 2 mm, repetition time (TR) = 6000 ms, echo time (TE) = Min Full, flip angle = 90°, number of excitations (NEX) = 2, acquisition matrix = 256 × 192, and field of view = 22 cm).

### Input function measurement

Dispersion and metabolite-corrected plasma input function was generated as described earlier [15]. In short summary: arterial blood was taken continuously at a rate of 2.5 ml/min for the first 22.5 min after radioligand injection and the blood radioactivity levels were measured using an automatic blood sampling system (Model # PBS-101 from Veenstra Instruments, Joure, The Netherlands). In addition, 4- to 8-ml manual arterial blood samples were obtained at 2.5, 7, 12, 15, 30, 45, 60, 90, and 120 min relative to time of injection [15]. A bi-exponential function was used to fit the blood-to-plasma ratios. A Hill function was used to fit the percentage of unmetabolized radioligand. The dispersion effect was modeled as to the convolution with a monoexponential with dispersion coefficient of 16 s and corrected with iterative deconvolution [20].

### Generation of ROI-based time activity curve

[^18^F]-FEPPA PET images were preprocessed and ROIs were automatically generated using in-house software, ROMI [19]. Briefly, ROMI fits a standard template of ROIs to an individual PD-weighted MR image based on the probability of gray matter, white matter, and cerebrospinal fluid (CSF). The individual MR images are then co-registered to each summed [^18^F]-FEPPA PET image using the normalized mutual information algorithm so that individual refined ROI template can be transferred to the PET image space to generate the time activity curve (TAC) for each ROI. Our a priori ROIs included cortical as well as subcortical brain regions such as frontal and temporal lobes, cingulate cortex, occipital lobe, insula, hippocampus, cerebellum, thalamus, caudate nucleus, and putamen.

Dynamical series of images of [^18^F]-FEPPA PET were visually checked for head motion and corrected using frame-by-frame realignment. Low-noise, nonattenuation-corrected images (created with iterative reconstruction) were used to optimize the frame-by-frame realignment process. A normalized mutual information algorithm was applied with SPM8 (Wellcome Trust Centre for Neuroimaging, London, UK) to co-register each frame to the frame that showed a high signal-to-noise ratio. Parameters from the normalized mutual information were applied to the corresponding attenuation-corrected dynamic images to generate a movement-corrected dynamic image. To address the potential issues of bias from the volume loss in older participants, time activity data for all participants was corrected for the effect of partial volume error (PVE) using the Mueller-Gartner partial volume error correction algorithm as implemented in Bencherif et al. [21].

### Kinetic analysis

*V*_T_ values in each ROI were derived from a two-tissue compartment model (2-TCM) using [^18^F]-FEPPA radioactivity in arterial plasma as an input function and a 5% vascular contribution [15]. This method has previously been validated for [^18^F]-FEPPA quantification [15, 22]. *V*_T_ is a ratio at equilibrium of the radioligand concentration in tissue to that in plasma (i.e., specific binding and non-displaceable uptake including non-specifically bound and free radioligand in tissue) and can be expressed in terms of kinetic rate parameters as *V*_T_ = *K*_1_/*k*_2_ (1 + *k*_3_/*k*_4_) where *K*_1_ and *k*_2_ are influx and efflux rates for radiotracer passage across the blood-brain barrier and *k*_3_ and *k*_4_ describe the radioligand transfer between the free and non-specific compartments and the specific binding compartment. We also measured the percentage of the coefficient of variation (%COV = 100% × standard error/mean), where standard error was estimated from the diagonal of the covariance matrix of nonlinear least-squares fitting [15]. From the different ROIs, we included *V*_T_ with %COV of ≤20, which assured less data noise. Therefore, the effective sample size for each ROI varied between 34 and 52. Kinetic analyses were performed using PMOD 3.6 modeling software (PMOD Technologies Ltd., Zurich, Switzerland).

### Voxel-based PET image analysis

For exploratory purposes, we investigated the difference between groups using voxel-based PET image analysis. Parametric images of [^18^F]-FEPPA *V*_T_ were generated using the Logan graphical analysis method [23]. A wavelet-based implementation of the kinetic modeling approach was applied to manage the low signal-to-noise ratio intrinsic of the voxel level quantification while maintaining the resolution [24, 25]. To examine voxel-wise group differences of *V*_T_, an independent sample *T* test was conducted using Statistical Parametric Mapping (SPM12-http://www.fil.ion.ucl.ac.uk/spm/software/spm12/). TSPO genotype (rs6971 polymorphism) was included as a covariate. Significant level for the whole brain analysis was thresholded at *p* < 0.001, uncorrected.

### DNA extraction and polymorphism genotyping

Genomic DNA was obtained from peripheral leukocytes using high salt extraction methods [26]. The polymorphism rs6971 was genotyped variously using a TaqMan1 assay on demand C_2512465_20 (AppliedBiosystems, CA, USA). The allele T147 was linked to Vic, and the allele A147 was linked FAM. PCR reactions were performed in a 96-well microtiter-plate on a GeneAmp PCR System 9700 (Applied Biosystems, CA, USA). After PCR amplification, end point plate read and allele calling was performed using an ABI 7900 HT (Applied Biosystems, CA, USA) and the corresponding SDS software (v2.2.2). Individuals with genotype Ala147/Ala147 were classified as HABs, Ala147/Thr147 as MABs, and Thr147/Thr147 as LABs [16]. [^18^F]-FEPPA is not quantifiable in LABs [12]; therefore, we did not include any LAB participants in our study. Generally, LAB participants represent less than 5% in a Caucasian sample [12, 18].

### Statistical analysis

Normality assumptions for all variables were assessed using the Kolmogorov-Smirnov test. Significant differences in means of normally distributed continuous demographical as well as clinical variables were tested by using factorial analysis of variance (ANOVA) and independent *t* tests. If variables did not satisfy the assumption, nonparametric Mann–Whitney *U* tests and Kruskal-Wallis tests were applied. Difference in proportions was assessed by chi-square tests. To assess any sub-group differences in *V*_T_ values, we used factorial ANOVA with TSPO genotype (HAB and MAB) and disease (PD and HC) as fixed factors in cortical as well as subcortical brain regions. We controlled for injected amount by adding it as a nuisance variable in all analyses, since there was a significant difference between HC and PD. A second level of analyses was performed using the independent *t* test to compare *V*_T_ values between HC-MABs and HC-HABs as well as PD-MABs and PD-HABs. Correlations between clinical measures and *V*_T_ values were investigated in all brain regions using Pearson’s correlation tests. All of the statistical analyses were performed using SPSS Statistics version 20.0. In order to establish significance, a threshold of *p <* 0.05 was applied.

## Results

### Demographic and clinical characteristics

Demographical and clinical measures are displayed in Table 1. Based on the rs6971 polymorphism, our study sample consisted of 25 MABs with 11 HC and 14 PD patients as well as 27 HABs with 11 HC and 16 PD patients.

**Table 1:** Demographics and clinical characteristics of study participants

	Total (*n =* 52)	HC (*n =* 22)	PD (*n =* 30)
Age, mean (SD)	65.0 (7.70)	64.4 (8.06)	65.5 (7.54)
Gender (M:F)	33:19	11:11	22:8
TSPO genotype	27 HAB, 25 MAB	11 HAB, 11 MAB	16 HAB, 14 MAB
Handedness (R:L:both)	38:5:1	13:0:1	25:5:0
Years of education, mean (SD)	16.4 (3.25)	16.3 (3.00)	16.4 (3.40)
MoCA, median (range)	28 (26–30)	28 (26–30)	28 (26–30)
MMSE, median (range)		29.5 (27–30)	
BDI II, median (range)	6 (0–23)	4.5 (0–16)	8 (0–23)
Symptom dominant side (R:L)			19:11
UPDRS III, median (range)			24 (4–45)
Duration of disease (years), median (range)			5 (2–15)
Total LEDD, mean (SD)			395.5 (483.21)
Amount injected (mCi), mean (SD)	4.9 (0.28)	4.8 (0.22)	5.0 (0.27)^a^
Mass injected (μg), mean (SD)	1.0 (0.70)	1.1 (0.83)	1.0 (0.59)
Specific radioactivity (mCi/μmol), mean (SD)	2670.8 (1559.56)	2707.8 (1626.74)	2643.6 (1536.00)

Values are expressed as mean (SD) and as median (range) where applicable

*UPDRS-III*, Unified Parkinson’s Disease Rating Scale III, *MoCA* Montreal Cognitive Assessment, *BDI II* Beck Depression Inventory II, *MMSE* Mini-Mental-State Examination, *LEDD* levodopa equivalent daily dose

^a^Significantly different

The factorial ANOVA showed that there were no significant differences in age as well as in specific activity at the time of injection or mass injected between MABs and HABs or between HC and PD groups. There was a significant difference in amount injected between HC (M = 4.79, SD = 0.22) and PD (M = 5.04, SD = 0.27, *t*(50) = −.3.59, *p =* 0. 001, two-tailed).

Chi-square tests showed that there were no significant differences in the composition of gender and handedness in the four groups or in the composition of symptom-dominant side between PD-MABs and PD-HABs. In addition, there were no significant differences in years of education, MoCA scores, MMSE scores, and BDI scores between MABs and HABs or between HC and PD groups. There were also no significant differences in UPDRS III, duration of disease, or total levodopa equivalent daily dose (LEDD) between PD-MABs and PD-HABs.

### Genotype and disease effects on TSPO binding

Levene’s tests of equality of error variances were significant only for following regions: frontal lobe (*p =* 0.016), caudate (*p =* 0.021), and putamen (*p =* 0.024). Therefore, we set a more stringent significance level of *p =* 0.01 for these brain regions.

**Fig. 1:**
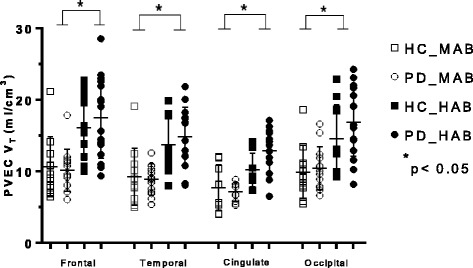
Graphs of partial volume effect corrected (PVEC) total distribution volume (*V*_T_) in different brain regions. Healthy control with mixed affinity binder (HC-MAB) and healthy control with high affinity binder (HC-HAB) groups as well as Parkinson’s disease with mixed affinity binder (PD-MAB) and Parkinson’s disease with high affinity binder (PD-HAB) groups. *Asterisks* indicate that the HAB groups show significantly higher *V*_T_ mean values compared with the MAB groups

**Fig. 2:**
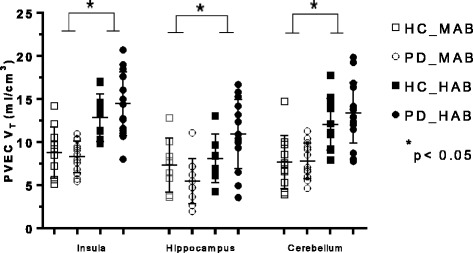
Graphs of partial volume effect corrected (PVEC) total distribution volume (*V*_T_) in different brain regions. Healthy control with mixed affinity binder (HC-MAB) and healthy control with high affinity binder (HC-HAB) groups as well as Parkinson’s disease with mixed affinity binder (PD-MAB) and Parkinson’s disease with high affinity binder (PD-HAB) groups. *Asterisks* indicate that the HAB groups show significantly higher *V*_T_ mean values compared with the MAB groups

**Fig. 3:**
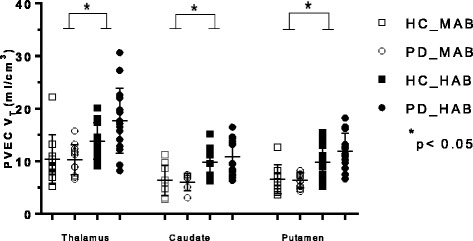
Graphs of partial volume effect corrected (PVEC) total distribution volume (*V*_T_) in different brain regions. Healthy control with mixed affinity binder (HC-MAB) and healthy control with high affinity binder (HC-HAB) groups as well as Parkinson’s disease with mixed affinity binder (PD-MAB) and Parkinson’s disease with high affinity binder (PD-HAB) groups. *Asterisks* indicate that the HAB groups show significantly higher *V*_T_ mean values compared with the MAB groups

There was a significant main effect of genotype on *V*_T_ values in every brain region (Table 2) as displayed in Figs. 1, 2, and 3. There was no main effect of disease in any region of interest as well as no disease × genotype interaction in any brain region (Table 2).

**Table 2:** Analysis of variance of regional TSPO *V*_T_ by diagnosis and TSPO genotype

	Factorial ANOVA				Independent *t* test					
	Disease effect^a^		Genotype effect^a^		HC group			PD group		
ROIs	*F* (df)	*p*	*F* (df)	*p*	HC-MAB (*n* = 11)	HC-HAB (*n* = 11)	%-difference	PD-MAB (*n* = 14)	PD-HAB (*n* = 16)	%-difference
Frontal	*F*_(1,46)_ = 0.0	0.964	*F*_(1,46)_ = 24.5	<0.001	13.9 (2.8)	19.4 (2.8)	33.0^b^	11.8 (0.2)	18.7 (1.6)	45.2^b^
Temporal	*F*_(1,47)_ = 0.0	0.846	*F*_(1,47)_ = 25.7	<0.001	11.9 (2.9)	16.1 (1.8)	30.0^b^	11.4 (1.2)	16.6 (1.2)	37.1^b^
Cingulate	*F*_(1,35)_ = 0.6	0.427	*F*_(1,35)_ = 23.0	<0.001	9.9 (1.6)	12.3 (1.5)	21.6	8.2 (0.0)	13.7 (1.1)	50.2^b^
Occipital	*F*_(1,46)_ = 1.3	0.257	*F*_(1,46)_ = 21.8	<0.001	12.3 (2.7)	17.2 (2.4)	33.2^b^	13.6 (3.0)	18.3 (1.4)	29.5^b^
Insula	*F*_(1,43)_ = 0.1	0.813	*F*_(1,43)_ = 34.6	<0.001	10.6 (1.8)	14.0 (1.6)	27.6^b^	9.8 (0.6)	15.7 (1.3)	46.3^b^
Hippocampus	*F*_(1,34)_ = 0.1	0.720	*F*_(1,34)_ = 7.8	0.009	8.7 (1.9)	10.4 (1.4)	17.8	8.6 (2.4)	12.4 (1.2)	36.2^b^
Cerebellum	*F*_(1,46)_ = 0.0	0.852	*F*_(1,46)_ = 34.4	<0.001	9.6 (2.2)	14.8 (1.6)	42.6^b^	9.2 (0.7)	14.7 (1.3)	46.0^b^
Thalamus	*F*_(1,43)_ = 0.6	0.445	*F*_(1,43)_ = 15.8	<0.001	12.8 (3.6)	16.8 (2.6)	27.0	13.5 (2.2)	19.9 (2.0)	38.3^b^
Caudate	*F*_(1,32)_ = 0.0	0.927	*F*_(1,32)_ = 14.6	0.001	8.1 (1.8)	12.4 (1.6)	41.6^b^	7.2 (0.3)	12.9 (1.0)	56.7^b^
Putamen	*F*_(1,41)_ = 0.1	0.717	*F*_(1,41)_ = 25.9	<0.001	8.0 (1.8)	13.5 (1.3)	51.2^b^	7.8 (0.4)	12.4 (1.1)	45.5^b^

Values are expressed as mean (SD). HAB, high affinity binders and MAB, mixed affinity binders refer to the single nucleotide polymorphism rs6971 of the TSPO gene known to influence [^18^F]-FEPPA binding. Percentage differences of the mean *V*_T_ values between HC-HABs and HC-MABs as well as PD-MABs and PD-HABs

^a^Main effect of univariate ANOVA

^b^Significantly different

Using independent *t* tests, when comparing the effect of genotype in the HC group, HC-HABs showed significantly higher *V*_T_ values in every brain region except the cingulate, hippocampus, and thalamus compared with the HC-MAB group. Similar results were observed among the PD group, whereas PD-HABs showed significantly higher *V*_T_ values in every brain region compared to PD-MABs (*p <* 0.05).

The percentage differences of the mean *V*_T_ values between HC-MABs and HC-HABs as well as PD-MABs and PD-HABs for each brain region are displayed in Table 2. The overall percentage difference of the mean FEPPA *V*_T_ between HC-MABs and HC-HABs was 32.6% (SD = 2.09) and for PD-MABs and PD-HABs was 43.1% (SD = 1.21).

There was no correlation between LEDD, UPDRS scores, and duration of disease with *V*_T_ values in any brain region in either PD-MABs or PD-HABs.

As an additional analysis, we also evaluated our data without PVE correction to identify any changes in the results. The main results were confirmed by showing no main effect of disease, or disease × genotype interaction as well as a main effect of genotype on *V*_T_ values (data not shown).

### Voxel-based PET image analysis

In accordance with the results of the ROI analysis, we did not find any significant difference between HC and PD groups, even when using an uncorrected threshold of *p <* 0.001.

## Discussion

This study represents an extension of our previous work investigating the potential use of [^18^F]-FEPPA as a new radioligand to measure neuroinflammation in PD patients. As previously reported [12, 18], we were able to observe an influence of the rs6971 polymorphism on TSPO binding affinity. Our results showed a trend towards elevated TSPO binding, especially in the PD-HAB group by showing significantly higher *V*_T_ values in every brain region compared to PD-MABs. *V*_T_ value comparisons between HC-HABs and HC-MABs were less consistent, by showing no significant *V*_T_ value differences in some subcortical and cortical brain regions. The trend towards elevated TSPO binding, particularly in PD-HABs was also previously observed in the striatum [18].

Currently, our results indicate interactions between the rs6971 polymorphism and neuroinflammation in PD. As mentioned earlier, TSPO expression may represent a potential in vivo biomarker of neuroinflammation. Increased TSPO expression has been observed after brain injury and inflammation in neurodegenerative diseases [7, 10, 27–29]. Earlier studies have proposed a connection between the rs6971 polymorphism and variations in pregnenolone production and plasma levels of low-density lipoprotein cholesterol [30], as well as anxiety in depressive individuals [31]. So far, the functional significance of the upregulated TSPO expression is still under investigation [9]. Besides its role as a potential biomarker for neuroinflammation, TSPO has many physiological functions such as its participation in cell growth and proliferation [32] and steroidogenesis [33], as well as in mitochondrial respiration and apoptosis [34]. Therefore, elevated TSPO levels in microglia and astrocytes may possibly increase neurosteroid synthesis at injury sites to stimulate neurotropic and neuroprotective activity [35]. Processes such as glial proliferation, migration, and phagocytosis or secretion of inflammatory cytokines in response to brain injury might be explanations for TSPO upregulation [35]. Nonetheless, since the genetic variability is present in healthy as well as in disease-affected brains, it cannot be associated with a specific neurological condition [12, 36]. However, previous investigations using second-generation TSPO radioligands have also presented a stronger disease effect on TSPO expression in AD patients [37]. Future investigations are needed to further determine the importance of this polymorphism and its clinical significance in PD.

Based on previous imaging studies from our lab [12, 38] and based on the mean and variability of *V*_T_ in our data (as displayed in Table 2), we used the *F*-test ANOVA to calculate the required total sample size, 52 participants will be needed to detect 30% difference between groups, assuming effect size *d* = 0.4, alpha = 0.05 and power = 0.8.

We were not able to show a significant main effect of disease on TSPO expression in any brain region. To date, several studies have investigated the association between neuroinflammation in PD using [^11^C]-PK11195 PET [10, 11, 29, 39–42]. The results have been inconclusive, as some studies were able to show increased neuroinflammation in PD patients compared to HC [10, 29, 39] and others not [11, 40]. This discrepancy might be explainable by the low specific to non-specific ratio of [^11^C]-PK11195 [12] and methodological differences. A recent study using [^11^C]-DPA713 PET revealed an extrastriatal spreading of microglial activation in a small sample of PD patients [43].

In our study, we used [^18^F]-FEPPA, a novel radioligand exhibiting optimal chemical, pharmacokinetic, and pharmacodynamic properties for applications in imaging TSPO [22]. Our previous results could not detect any significant disease effect on [^18^F]-FEPPA *V*_T_ in the striatum [18]. In our current investigation, we were not able to show any anatomically widespread microglial activation in Parkinson disease, as previously observed using [^11^C]-PK11195 [29]. To this point, several studies observed a significant increase in [^18^F]-FEPPA *V*_T_ in people with major depressive episodes [44] as well as AD patients [45]. Especially in AD patients, a widespread distribution of activated microglia was noticed in gray and white matter indicating an important role of neuroinflammation in cognitive decline [45]. In addition, several studies [41, 42] including more severe cases of PD patients with dementia were able to show neuroinflammation in primarily cortical regions. Since our study sample consisted of cognitive normal PD patients a significant disease effect might be detectable in more cognitively impaired patients.

We did not find any correlation between anti-parkinsonian medication, disease severity and duration, and [^18^F]-FEPPA *V*_T_. Similar observations were detected in longitudinal data [29], showing no correlation between levels of microglial activation and clinical severity as well as disease duration. The authors of this study suggest that microglia are activated early in the disease process, but remain relatively static over a 2-year time interval. Further, in consistency with previous results [18, 46] using the same radioligand, we did not observe any age-related increase in TSPO density (data not shown).

## Conclusions

In conclusion, more studies are needed to determine the significance of [^18^F]-FEPPA as a biomarker of neuroinflammation in PD and to further explore its capacity to differentiate between PD patients with cognitive impairment or even dementia.

## Abbreviations

2-TCM: Two-tissue compartment model
AD: Alzheimer disease
ANOVA: Analysis of variance
BDI: Beck Depression Inventory
COV: Coefficient of variation
CSF: Cerebrospinal fluid
FOV: Field of view
HAB: High-affinity binder
HC: Healthy control
HRRT: High Resolution Research Tomography
LAB: Low-affinity binder
LEDD: Levodopa equivalent daily dose
MAB: Mixed-affinity binder
MMSE: Mini-Mental State Examination
MoCa: Montreal Cognitive Assessment
NEX: Number of excitations
PD: Parkinson’s disease
PD-weighted: Proton density-weighted
PET: Positron emission tomography
PVE: Partial volume error
PVEC: Partial volume error correction
ROI: Region of interest
SD: Standard deviation
TAC: Time activity curve
TE: Echo time
TR: Repetition time
TSPO: Translocator protein
UPDRS: Unified Parkinson Disease Rating Scale
*V*_T_: Total distribution volume
